# HUNK Signaling in Metastatic Breast Cancer

**DOI:** 10.18632/oncoscience.504

**Published:** 2020-05-05

**Authors:** Tinslee Dilday, Nicole Ramos, Elizabeth Yeh

**Affiliations:** ^1^Department of Pharmacology and Toxicology, Indiana University School of Medicine, Melvin and Bren Simon Comprehensive Cancer Center, Indianapolis, IN, USA; ^*^These authors contributed equally.

**Keywords:** HUNK, breast cancer, metastasis, EGFR, phosphorylation

## Abstract

Once metastatic disease has occurred, there is no cure for breast cancer. Consequently, identifying factors that promote and support breast cancer metastasis is critical for understanding how to pharmacologically target this process. Hormonally up-regulated neu-associated kinase (HUNK) is a serine/threonine protein kinase related to the sucrose non-fermenting-1 (Snf-1)/5’ adenosine monophosphate-activated protein kinase (AMPK) family of kinases. HUNK has been found to play a role in breast cancer metastasis. However, conflicting reports indicate HUNK is a metastasis promoting factor as well as an inhibiting factor. Our group recently provided evidence that supports the conclusion that HUNK is a metastasis promoting factor by showing that HUNK regulates breast cancer metastasis through phosphorylation of EGFR. Here, we summarize our findings and discuss their implications toward pharmacological targeting of HUNK in breast cancer.

Hormonally Up-regulated Neu-associated Kinase (HUNK) is a serine-threonine protein kinase that is a member of the Snf-1/AMPK protein kinase family [1]. HUNK is also known as metastasis associated protein kinase in VMR tumor family (MAK-V) [2]. Recently described functions and biological roles for HUNK include the regulation of cell survival, cell proliferation, and cell metabolic regulation [3-7]. Consequently, HUNK has been shown to play a role in human cancers due to its ability to regulate these cellular processes. In particular, a role for HUNK in breast cancer is evident, given that this kinase was originally isolated from the mouse mammary gland [1]. Prior studies show HUNK expression is regulated by Human Epidermal Growth Factor Receptor 2 (HER2) oncogene activation in breast cancer and that HUNK expression is important for tumor cell survival in the HER2-positive (HER2+) subtype of breast cancer [6, 8]. In the HER2+ subtype, HUNK regulates the cell survival pathways, autophagy and apoptosis, to promote tumor cell survival and therapeutic resistance to HER2 inhibitors [4, 6, 9]. Additional studies show that HUNK plays a role in breast cancer metastasis, likely in the triple-negative subtype [10-12]. Interesting, HUNK is reported to participate in metastasis through mechanisms that are independent from its activity in the HER2+ breast cancer subtype. Here, we discuss HUNK’s role in metastasis by reviewing past publications and discussing our current findings, including kinase dependent mechanisms for HUNK in driving breast cancer metastasis.

In a 2009 publication, Wertheim et. al. reported that HUNK promotes mammary tumor metastasis using a MMTV-myc mammary tumor model [11]. The study authors crossed the MMTV-myc mouse model into a *Hunk* knockout (*Hunk^-/-^*) background and evaluated the resulting mice for the formation of spontaneous metastasis. They found that MMTV-myc;*Hunk^-/-^* mice had reduced lung metastasis compared to MMTV-myc;Hunk wildtype (*Hunk^+/+^*) mice. The study authors then generated tumor cell lines from mammary tumors isolated from the MMTV-myc;*Hunk^+/+^* and MMTV-myc;*Hunk^-/-^* mice. Tumor cells from the MMTV-myc;*Hunk^-/-^* mice were further engineered to express either a wildtype (WT) Hunk or a kinase inactive Hunk (K91M). When tested in transwell migration and invasion assays, MMTV-myc;*Hunk^-/-^*, WT Hunk expressing cells migrated more than MMTV-myc;*Hunk^-/-^*, K91M Hunk cells. Furthermore, the study authors demonstrate that re-expression of K91M Hunk in MMTV-myc;*Hunk^-/-^* derived metastatic cell lines failed to rescue metastasis by orthotopic xenograft assay, compared to when WT Hunk was re-expressed. Taken together, these results suggest that Hunk/HUNK kinase activity is important for the metastatic process. However, this study did not identify direct substrates of Hunk/HUNK that were dependent on kinase activity for metastasis.

In contradiction to this first publication, a second paper published in 2010 by Quintela-Fandino et. al. reported that HUNK suppresses metastasis in basal type breast cancers [12]. This group used the human basal-type breast cancer cell lines, MDA MB 468 and MDA MB 231 cells, to show that exogenous expression of HUNK in these cell lines suppresses transwell invasion as well as in vivo metastasis. The study authors describe a mechanism whereby HUNK binds to cofilin-1 (CFL-1) to prevent the dephosphorylation of CFL-1 by protein phosphatase 2A (PP2A), maintaining CFL-1 in a phosphorylated state. However, HUNK does not directly phosphorylate CFL-1. Since CFL-1 regulates actin cytoskeleton polymerization and CFL-1 is inactive toward this process when it is phosphorylated, the study authors go on to evaluate the effects of HUNK on cytoskeleton rearrangement in response to EGF stimulation [13]. Here, the authors engineered MDA MB 468 cells to express a vector control or to overexpress WT HUNK, and then treated each of these cell populations with EGF. The effects of EGF stimulation on cytoskeletal rearrangement were then evaluated and the authors found that WT HUNK expressing MDA MB 468 cells lacked the ability to undergo cytoskeletal rearrangement in response to EGF. This study neither presented a kinase dependent role for HUNK in metastasis nor identified a HUNK specific substrate that was responsible for these activities.

The two aforementioned studies differed in their approach to evaluating breast cancer metastasis. The former made use of an established genetically engineered mouse mammary tumor model (GEMM) and took an approach to deplete *Hunk* by gene knockout, while the latter focused on using human breast cancer cell lines and took an approach to overexpress HUNK [12, 14]. The argument was made that the human cell lines in the latter publication represent “basal” type breast cancer and that this difference could account for the discrepancies between the studies. However, gene expression studies suggest that the MMTV-myc GEMM is consistent with the human triple-negative breast cancer subtype, which includes basal type breast cancers [15, 16].

Consistent with the 2009 study from Wertheim et. al., our findings showed that HUNK is pro-metastatic. Separate from the two prior studies, our study showed that HUNK phosphorylates the epidermal growth factor receptor (EGFR) at threonine (T) 654 to regulate breast cancer metastasis, providing a discrete HUNK-kinase dependent mechanism for this process [10]. Our study used the 4T1 mouse mammary tumor cell line as well as two human basal breast cancer cell lines, the MDA MB 468 and BT20 model. Therefore, we tested a mouse and two human models. We took an approach to deplete *Hunk*/HUNK in these cell lines using shRNA targeting. We also used a pharmacological inhibitor of HUNK as a tool compound to modulate kinase activity [17]. In all three of these cell lines, HUNK depletion or inhibition led to an impairment of cell migration and invasion that was concomitant with a loss of EGFR phosphorylation at T654. Furthermore, HUNK downregulation or inhibition resulted in a decrease in mesenchymal marker expression and an increase in epithelial marker expression, suggesting that HUNK activity correlates with epithelial to mesenchymal transition (EMT). We also showed these effects in vivo using the 4T1 model where mice that harbored mammary tumors from *Hunk*-depleted cells had a markedly reduced number of spontaneous lung metastases develop compared to mice with tumors derived from control cells expressing a non-functional shRNA. Tumors that formed from the *Hunk*-depleted cells also showed a loss of EGFR phosphorylation at T654. Based on these findings, we concluded that HUNK promotes breast cancer metastasis (Figure 1).

While our technical approaches did not exactly replicate either of the previous studies, we noted that our study used MDA MB 468 cells similar to the 2010 study by Quintela-Fandino et. al. However, our conclusions from the use of the MDA MB 468 cell line differed from this particular study. We showed that shRNA depletion of HUNK reduced cell migration and invasion compared to cells expressing a non-functional shRNA control, whereas the prior study showed that overexpression of HUNK suppresses migration and invasion [10, 12]. While the use of individual reagents and techniques could certainly be attributed to the differences in results, another possibility is that exogenous HUNK could act as a dominant negative toward endogenous protein. We also noted that our study did not evaluate CFL-1 phosphorylation or EGF modulation of actin cytoskeleton. We also did not re-introduce either a WT HUNK or K91M HUNK into our HUNK-depleted cells, therefore, these areas remain to be tested.

Our study significantly differed from either of the prior studies because we identified a direct substrate of HUNK that provided a mechanism for the metastatic behavior of the mammary tumor and breast cancer cell lines we tested. To date, very few HUNK substrates are identified [7, 10, 18, 19]. Three of the proteins identified as HUNK substrates; EGFR, Run domain Beclin-1-interacting and cysteine-rich domain-containing protein (RUBICON), and integral membrane protein 2A (ITM2A), are described in the context of breast cancer [7, 10, 19]. A fourth protein phosphorylated by HUNK is dishevelled (Dsh), which has critical roles in eye and brain development [18]. This study was carried out using *Xenopus laevis*. Interestingly, developmental defects in eye and brain, or any other organ system, have not been reported in mouse models where *Hunk* is either genetically deleted or transgenically overexpessed [14, 20, 21]. Consequently, additional investigation is needed to clarify these findings.

Future studies to evaluate known HUNK substrates, as well as continuing to identify new HUNK substrates, will greatly facilitate our understanding about how HUNK participates in the pathological development of human disease. Moreover, once a clinically relevant pharmacological inhibitor is developed for HUNK, the knowledge generated about HUNK substrates will greatly facilitate the pharmacological study of HUNK within models of human disease.

**Figure 1:**
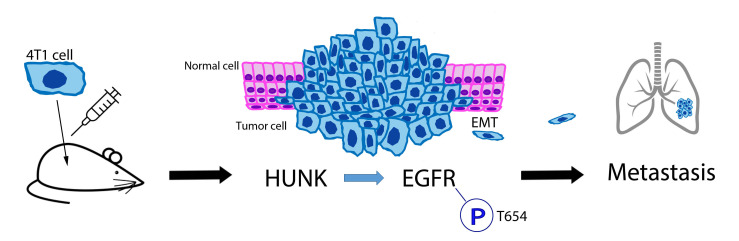
4T1 in-vivo mouse model shows the pro-metastatic nature of HUNK in breast cancer. 4T1 breast cancer cells were injected in the mammary glands of mice then monitored for their tumor growth and metastasis. It was found that HUNK’s direct phosphorylation of EGFR at T654 promotes epithelial mesenchymal transition, leading to cancer metastasis.
